# Effects and relationships of early physiotherapy on respiratory functions and functional capacity at discharge in patients undergoing open heart surgery

**DOI:** 10.1186/s12872-026-05674-3

**Published:** 2026-03-04

**Authors:** Yasemin Şahbaz, İpek Yeldan, Tülin Özalhas, Doğan Yetüt, İbrahim Ufuk Alpagut

**Affiliations:** 1 Department of Physiotherapy and Rehabilitation, Faculty of Health Sciences, İstanbul Beykent University, Istanbul, Turkey; 2 Department of Physiotherapy and Rehabilitation, Faculty of Health Sciences, İstanbul University-Cerrahpaşa, Istanbul, Turkey; 3 Department of Cardiovascular Surgery, Istanbul Faculty of Medicine, İstanbul University, Istanbul, Turkey; 4 Department of Cardiovascular Surgery, İstanbul Beykent University, Istanbul, Turkey

**Keywords:** Cardiac rehabilitation, Functional capacity, Open heart surgery, Respiratory muscle strength

## Abstract

**Background:**

Open heart surgery (OHS) often leads to declines in respiratory functions and functional capacity. Postoperative challenges, including changes in pulmonary function and respiratory muscle strength, can negatively impact patients’ recovery and quality of life. Early physiotherapy interventions aim to support the restoration of respiratory and physical functions during the critical discharge phase. The aim of this study was to investigate the changes in pulmonary function, respiratory muscle strength, and functional capacity, as well as the relationships among these markers, in patients discharged after undergoing open heart surgery.

**Methods:**

The pre-operative and post-operative discharge stage assessment results of 34 patients were examined. The evaluations included in the medical records consisted of demographic data, respiratory functions (spirometric measurements reported as % predicted and intraoral pressure measurements), and functional capacity measurement results.

**Results:**

It was found that patients’ Forced Vital Capacity (FVC) (% predicted) and 6-Minute Walk Test (6MWT) distance at discharge were significantly reduced compared to pre-operative values. Forced Expiratory Volume in One Second (FEV_1_) (% predicted), however, showed no statistically significant change. After applying Benjamini-Hochberg False Discovery Rate (FDR) correction, significant positive correlations were observed between FVC (% predicted) and Forced Expiratory Flow at 25–75% of Vital Capacity (FEF25-75) (% predicted); FVC (% predicted) and Peak Expiratory Flow (PEF) (% predicted); PEF (% predicted) and FEF25-75 (% predicted); PEF (% predicted) and Maximal Expiratory Pressure (MEP); FEV_1_ (% predicted) and 6MWT; Maximal Inspiratory Pressure (MIP) and MEP; and MIP and 6MWT.

**Conclusions:**

In patients undergoing OHS who received early physiotherapy, FVC and 6MWT distance significantly declined at discharge compared to preoperative levels, while PEF, FEV_1_, FEF25-75, MIP, and MEP did not show significant changes. The decreased FVC and FEV_1_ at discharge were specifically related to functional capacity. Therefore, routine assessment of respiratory muscle strength is essential in OHS patients, with results needing evaluation within homogeneous surgical groups to guide effective rehabilitation.

**Clinical trial registration:**

This study was registered at ClinicalTrials.gov (Identifier NCT05932368) on June 20, 2023.

## Introduction

Pulmonary complications are a common occurrence following open-heart surgery, with the potential to negatively impact patients’ respiratory functions and prolong the recovery process. These complications include serious conditions such as hypoxemia, atelectasis, pleural effusion, diaphragmatic dysfunction, and acute respiratory failure, all of which have been shown to increase postoperative morbidity and mortality rates [1]. Respiratory mechanics are significantly affected after surgery, leading to a notable decrease in lung volume, disruption of gas exchange processes, and alterations in the ventilation/perfusion ratio. Consequently, there is a marked reduction in cardiorespiratory capacity [2]. Respiratory functions and respiratory muscle strength are subject to a multitude of influences, resulting in considerable inter-individual variability. In particular, the inability to extubate patients within the first 24 h post-surgery and prolonged mechanical ventilation play a pivotal role in increasing morbidity rates and negatively impacting pulmonary functions. Prolonged extubation times lead to longer periods of intensive care unit admission and extended hospitalizations [3]. It is observed that nearly all patients who have undergone heart surgery experience measurable impairments in their respiratory functions. Furthermore, approximately 10–25% of these patients develop significant health issues, such as postoperative pulmonary complications. A variety of factors, including those occurring preoperatively, intraoperatively, and postoperatively, influence patients’ capacity to manage these complications [4].

In the postoperative period, factors such as prolonged bed rest, thoracic incision, extended cardiopulmonary bypass, preoperative dyspnea, and physical impairment, along with postoperative complications and sternal healing limitations, can negatively impact both respiratory functions and exercise capacity [5]. Patients undergoing open-heart surgery often experience a significant decline in functional capacity. Consequently, cardiac rehabilitation is of paramount importance during the postoperative period [6]. Cardiac rehabilitation is a multifaceted therapeutic modality aimed at enhancing the functional capabilities and health-related quality of life of individuals with cardiovascular disease. The benefits of cardiac rehabilitation have been demonstrated to be consistent, regardless of the treatment method or setting. The objective of these rehabilitation programs is to promote a more rapid improvement in functional capacity and reduce the risk of complications [5].

While numerous studies separately evaluate respiratory functions, respiratory muscle strength, and functional exercise capacity in patients undergoing cardiac rehabilitation after OHS, few studies examine all these factors together and investigate their relationships during the preoperative and discharge phases. In this context, our study aims to investigate the early changes and relationships between respiratory functions, respiratory muscle strength, and functional capacity in patients who have undergone OHS.

## Methods

This retrospective study examined patient data from individuals who underwent open-heart surgery at the Department of Cardiovascular Surgery, Istanbul University Istanbul Faculty of Medicine. Patient records were selected from those with complete discharge documents from 2018 to 2022. A total of 34 patients were included in the study after obtaining necessary data use permissions. Data pertaining to the preoperative and postoperative discharge assessments were retrieved from the patient records.

The study was conducted in accordance with the ethical standards set forth in the Helsinki Declaration and was approved by the Clinical Research Ethics Committee of Istanbul University Istanbul Faculty of Medicine (approval number: 1208327; date: 09/15/2022). After being contacted, all patients were informed about the study both verbally and in written form, and written informed consent was obtained.

### Participants

The inclusion criteria for the study were as follows: patients aged between 25 and 70 years, who had undergone open-heart surgery, and were enrolled in the phase 1 cardiac rehabilitation program conducted by a researcher physiotherapist. Additionally, patients were required to have a preoperative ejection fraction exceeding 40%. Patients whose records did not meet the inclusion criteria, had incomplete data, or did not provide the necessary permission for data use were excluded from the study.

### Data collection and measurements

lthough not part of the department’s routine practice, spirometric measurements, respiratory muscle strength assessments, and functional capacity evaluations were conducted by two different physiotherapists who were blinded to the patients’ assessment stage (pre-operative or discharge) during the assessment period. The dataset comprised demographic information, respiratory function data (spirometric measurements and oral pressure measurements), and functional capacity assessment results.

### Spirometric measurements

For spirometry, the highest results from three repeated measurements were recorded and evaluated according to the criteria established by the American Thoracic Society/European Respiratory Society (ATS/ERS). The parameters assessed included FVC, FEV1, PEF, and FEF25-75. Spirometry was performed using a Medical International Research Spirodoc^®^ device, which was calibrated and quality-controlled. All spirometric values were expressed as percentages of predicted normal values (% predicted) calculated automatically by the device software based on established reference equations appropriate for the patients’ age, sex, height, and ethnicity, in accordance with ATS/ERS guidelines [7].

### Oral pressure measurement

For respiratory muscle strength testing, the highest results from three repeated measurements were recorded for MIP and MEP and evaluated according to ATS/ERS criteria. Respiratory muscle strength was assessed using a portable, electronic oral measurement device, the MPM 100 MEC-Belgium, which was calibrated and quality-controlled. To interpret the results, normal values for inspiratory and expiratory muscle strength, stratified by age and sex, were utilized, and the resulting percentages were calculated relative to these expected values [8].

### Functional capacity

The functional capacity of the subjects was assessed using the 6MWT. This test was conducted in a 30-meter long hospital corridor, with the distance walked in six minutes recorded in accordance with American Thoracic Society (ATS) guidelines [9].

### Phase 1 cardiac rehabilitation program

The following summarizes the key aspects of the program, which is conducted under the supervision of a physiotherapist:

- Initial 24-Hour Period: The patient is placed on bed rest and closely observed.
- Extubated Patients: Focus on the awakening and orientation of patients who have undergone extubation.
- Monitoring: Continuous monitoring of vital signs, including blood pressure, heart rate, and respiratory rate.
- Patient Education: Patients received education regarding their condition and rehabilitation process.
- Hemodynamic Stability: The stability of the patient’s hemodynamic state is paramount, and is monitored before, during, and after interventions.
- Exercise Termination: Exercise should be stopped in the event of severe dyspnea, significant fatigue, dizziness, fever, arrhythmia, tachypnea, tachycardia, or other signs of instability, until the patient’s condition has stabilized.
- Type of Exercise: Unilateral exercises should be avoided.
- Session Duration: Each session should last between 45 and 60 min.
- Session Frequency: The program is performed once daily.
- Timing of Sessions: Sessions are conducted in the afternoon. The specifics of the treatment program are outlined in Table 1.
  **Table 1:** Phase 1 Cardiac Rehabilitation Protocol

Day	Key Activities
Day 1	Patient orientation. breathing exercises. postural drainage. huffing exercises. passive & active joint movements. seated position training (1-10 min).
Day 2	Increased repetitions (10 reps). assisted/active joint movements. active breathing cycle. sitting for 15 min (3 sessions). initial standing attempt.
Day 3	Increased chest physiotherapy & sitting duration. postural exercises. head & shoulder mobilization. walking initiation (5-10 meters).
Day 4	Walking distance progression (30-60 meters).
Day 5	Walking extended to 80-100 meters. monitored standing exercises for fatigue.
Day 6	Gradual walking distance increase. stair training (max 10 steps).
Day 7-10	Continued program if patient remains hospitalized. stair training extended (10-12 steps).

### Statistical analysis

Data analysis was conducted using SPSS version 25. The distribution of participants by gender, presence of chronic diseases, regular medication use, smoking habits, alcohol consumption, and regular exercise habits was evaluated using the chi-square test. The normality of the data was tested using the Shapiro-Wilk test. The differences between preoperative and postoperative measurements were assessed using the paired sample t-test. Pearson correlation test was employed to analyze relationships between independent variables. To account for multiple comparisons in the correlation analysis, Benjamini-Hochberg FDR correction was applied to control for multiple comparisons while maintaining statistical power and reducing the risk of Type I errors. Additionally, paired-samples t-tests were used to assess the statistical significance of the difference between MIP and MEP values, separately for the preoperative and discharge time points, to identify potential differential impairment of respiratory muscle groups.

## Results

In this retrospective study, we analyzed the records of 34 patients who underwent open-heart surgery and participated in a Phase 1 cardiac rehabilitation program under physiotherapist supervision. Patients’ demographic and clinical characteristics are presented in Table 2.

**Table 2:** Demographic and clinical characteristics of the patients

Evaluations	Mean ± SD (%)	Evaluations	Mean ± SD (%)
Age (years)	61±6	Comorbidity Score (0-37)	4±1
Gender (F/M)	11/23 (32/68)	Ejection Fraction	55±8
BMI (kg/m²)	27±5	Disease Duration (months)	58±65
Education Level-Illiterate-Primary School-Middle School-University-Postgraduate	6(18)17 (50)9 (27)1 (3)1 (3)	Type of Surgery-CABG-Valveo MVR-ASD-Valve+Bypass	21 (62) 11 (32)1 (3)1 (3)
Marital Status-Married-Single-Other	24 (71)3 (9)7 (21)	Exercise-Yes-No	2 (6)32 (94)
Smoking Status-Smoker-Non-smoker-Former Smoker	22 (645)11 (32)1 (3)	Alcohol Consumption-Drinks-Does not drink-Quit	8 (24)24 (71)2 (6)
Duration on Pump (Minutes)	118±22	Intubation Duration (hours)	9±4
ICU Stay (Days)	3±1	Ward Stay (days)	6±1
Total Hospital Stay (Days)	9±2	Total Number of Patients	34

*SD* Standard Deviation, *BMI* Body Mass Index, *F* Female, *M *Male, *ICU* Intensive Care Unit, *CABG*  Coronary Artery Bypass Graft, *MVR* Mitral Valve Replacement, *ASD* Atrial Septal Defect

**Fig. 1:**
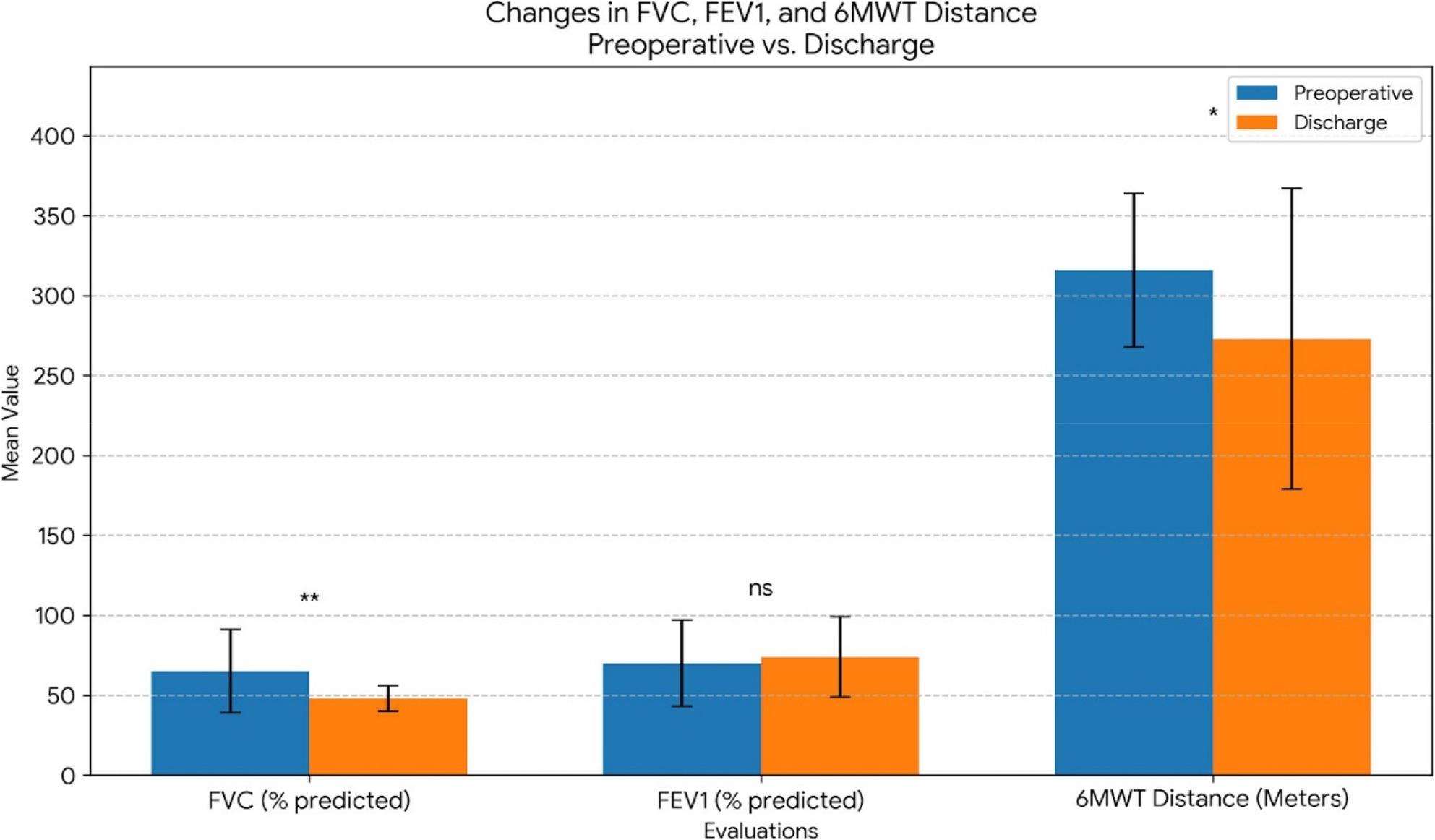
Changes in FVC, FEV1, and 6MWT Distance Preoperative vs. Discharge. Changes in FVC (% predicted), FEV1 (% predicted), and 6-Minute Walk Test (6MWT) Distance between Preoperative and Discharge Periods. Values are presented as Mean ± Standard Deviation (SD). *** indicates *p* < 0.001, * indicates *p* < 0.05, ns indicates non-significant (*p* ≥ 0.05)

We found that patients’ FVC and 6MWT distance at discharge significantly decreased compared to preoperative levels. Specifically, FVC decreased by approximately 26% (*p* = 0.001), and 6MWT distance by approximately 14% (*p* = 0.010). FEV_1_, however, showed a non-significant increase of approximately 5.7% from 70 ± 27% preoperatively to 74 ± 25% at discharge (*p* = 0.529). Additionally, the FEV_1_/FVC ratio was 77 ± 11% preoperatively and 82 ± 18% at discharge, with no statistically significant change (*p* = 0.058). Table 3 details the mean values of the data examined preoperatively and at discharge, as well as the changes between these values (as visually represented in Fig. 1).

As detailed in Table 3, while we observed no significant changes in mean MIP and MEP values between the preoperative period and discharge (showing approximately a 5% decrease for MIP and a 4% increase for MEP), a distinct pattern of differences was noted when comparing MIP and MEP values within each time point. Specifically, MEP values were significantly higher than MIP values both preoperatively (*p* = 0.001) and at discharge (*p* < 0.001). This finding suggests a potential differential impact on inspiratory versus expiratory muscle strength following open-heart surgery.

**Table 3:** Preoperative and discharge respiratory functions. respiratory muscle strength. and functional capacity values of the patients

Evaluations	PreoperativeMean ± SD	DischargeMean ± SD	p-value	Cohen’s d	95% CI
FVC (% predicted)	65±26	48±8	0.001^a^	0.93	(10.02-24.74)
FEV_1_/FVC	77±11	82±18	0.058^a^	-0.34	(−1.15−8.63)
PEF (% predicted)	46±27	37±15	0.051^a^	0.42	(-0.61−20.25)
FEV_1_ (% predicted)	70±27	74±25	0.529^a^	-0.15	(−4.87−16.39)
FEF_25-75_(% predicted)	41±21	35±11	0.071^a^	0.37	(-0.56−14.04)
MIP^b^	39±18	37±14	0.360^a^	0.12	(-2.05−7.87)
MEP^b^	53±24	55±17	0.574^a^	-0.10	(-1.23−10.57)
6MWT Distance (Meters)	316±48	273±94	0.010^a^	0.57	(15.21−83.03)

*SD* Standard Deviation, *CI* Confidence Interval, *FVC* Forced Vital Capacity, *FEV1* Forced Expiratory Volume in One Second, *PEF* Peak Expiratory Flow, *FEF25-75* Forced Expiratory Flow at 25-75% of Vital Capacity, *MIP* Maximal Inspiratory Pressure, *MEP* Maximal Expiratory Pressure, *6MWT* 6-Minute Walk Test

a.        Paired-samples t-test comparing Preoperative vs. Discharge values for each variable. p<0.05

Paired-samples t-test comparing MIP vs. MEP within the same time point: - Preoperative MIP vs. Preoperative MEP: *p* = 0.001 - Discharge MIP vs. Discharge MEP: *p* < 0.000001 (or *p* < 0.001)

**Fig. 2:**
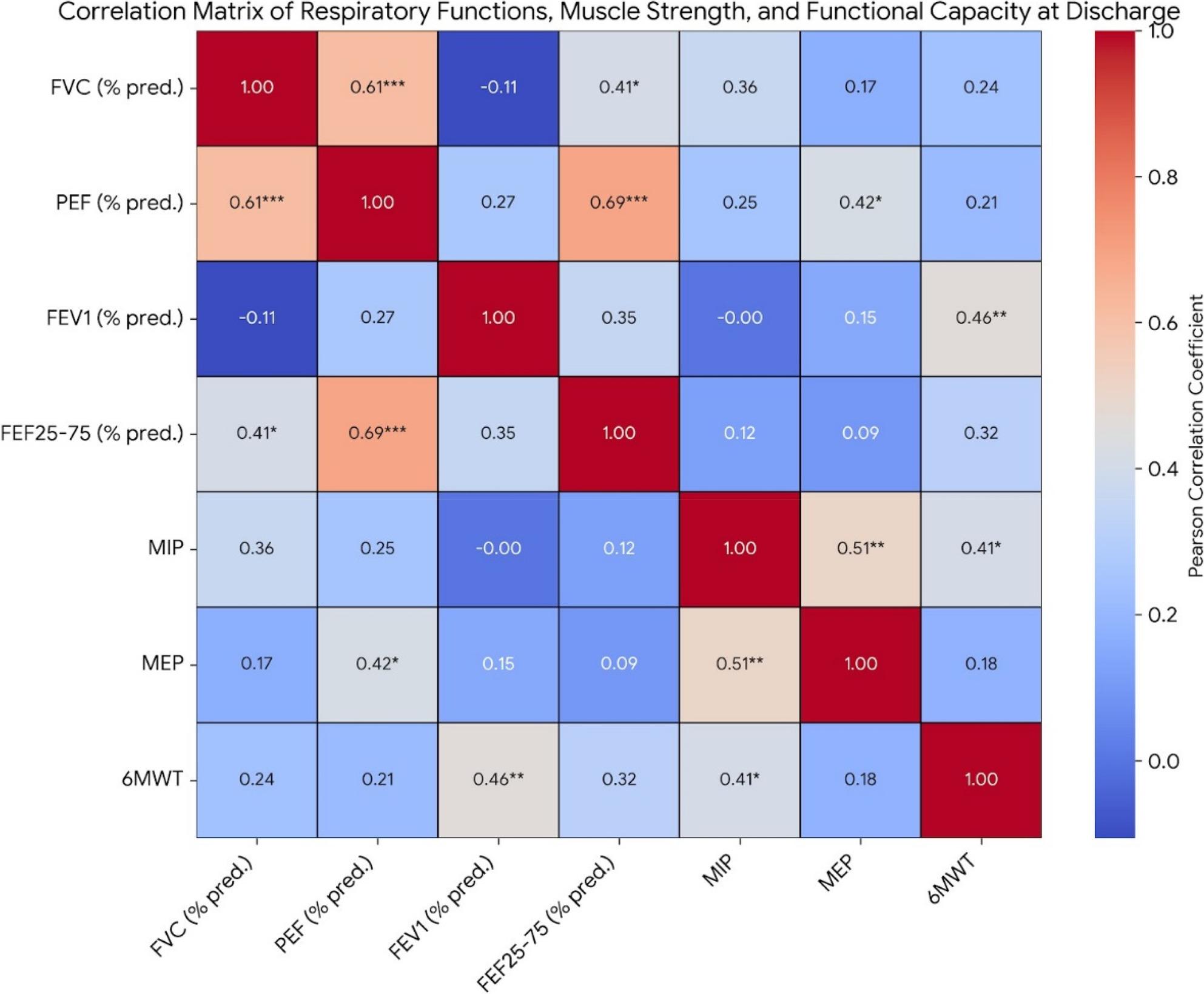
Correlation Matrix of Respiratory Functions, Respiratory Muscle Strength, and Functional Capacity Indicators at Discharge. Correlation Matrix of Respiratory Functions, Respiratory Muscle Strength, and Functional Capacity Indicators at Discharge. Values represent Pearson Correlation Coefficients (r). * indicates p < 0.05, ** indicates p < 0.01, *** indicates p < 0.001. FVC: Forced Vital Capacity; FEV1: Forced Expiratory Volume in One Second; PEF: Peak Expiratory Flow; FEF25-75: Forced Expiratory Flow at 25-75% of Vital Capacity; MIP: Maximal Inspiratory Pressure; MEP: Maximal Expiratory Pressure; 6MWT: 6-Minute Walk Test

As detailed in Table 4, after applying Benjamini-Hochberg FDR correction, several significant correlations were observed (Fig. 2). Specifically, FVC showed a strong positive correlation with PEF (*r* = 0.612, *p* < 0.001) and a significant positive correlation with FEF25-75 (*r* = 0.409, *p* = 0.045). No significant correlation was observed between FVC and FEV_1_ (*r*=-0.106, *p* = 0.550). FEV_1_ demonstrated a strong positive correlation with 6MWT (*r* = 0.460, *p* = 0.006), but no significant correlations were found between FEV_1_ and PEF (*r* = 0.267, *p* = 0.126) or FEV_1_ and FEF25-75 (*r* = 0.353, *p* = 0.093). PEF demonstrated a strong positive correlation with FEF25-75 (*r* = 0.692, *p* < 0.001) and a significant positive correlation with MEP (*r* = 0.417, *p* = 0.045). MIP showed a strong positive correlation with MEP (*r* = 0.505, *p* = 0.002) and a significant positive correlation with 6MWT (*r* = 0.411, *p* = 0.016).

**Table 4:** Correlation between respiratory functions. respiratory muscle strength. and functional capacity indicators at discharge

Evaluations	FVC (% pred.), r/p	PEF (% pred.), r/p	FEV_1_ (% pred.),r/p	FEF_25-75 _(% pred.),r/p	MIP, r/p	MEP, r/p	6MWT, r/p
FVC (% pred.)	1	.612^**^/0.000 (0.30 - 0.75)	-0.106/0.550 (-0.45 - 0.28)	.409^*^/0.045 (0.02 - 0.78)	.362/0.092 (-0.05 - 0.72)	.174/0.326 (-0.32 - 0.63)	.239/0.174 (-0.21 - 0.68)
PEF (% pred.)	.612^**^/0.000 (0.30 - 0.75)	1	.267/0.126 (-0.19 - 0.63)	.692^**^/0.000 (0.40 - 0.88)	.249/0.155 (-0.12 - 0.59)	.417^*^/0.045 (0.02 - 0.76)	.211/0.231 (-0.23 - 0.62)
FEV_1_ (% pred.)	-0.106/0.550 (-0.45 - 0.28)	.267/0.126 (-0.19 - 0.63)	1	.353/0.093 (-0.09 - 0.71)	-0.001/0.996 (-0.50 - 0.49)	.153/0.386 (-0.27 - 0.58)	.460^**^/0.006 (0.15 - 0.79)
FEF_25-75_(% pred.)	.409^*^/0.045 (0.02 - 0.78)	.692^**^/0.000 (0.40 - 0.88)	.353/0.093 (-0.09 - 0.71)	1	.121/0.494 (-0.31 - 0.55)	.094/0.599 (-0.35 - 0.53)	.315/0.070 (-0.05 - 0.65)
MIP	.362/0.092 (-0.05 - 0.72)	.249/0.155 (-0.12 - 0.59)	-0.001/0.996 (-0.50 - 0.49)	0.121/0.494 (-0.31 - 0.55)	1	.505^**^/0.002 (0.25 - 0.82)	.411^*^/0.016 (0.10 - 0.73)
MEP	.174/0.326 (-0.32 - 0.63)	.417^*^/0.014 (0.02 - 0.76)	.153/0.386 (-0.27 - 0.58)	.094/0.599 (-0.35 - 0.53)	.505^**^/0.002 (0.25 - 0.82)	1	.181/0.305 (-0.29 - 0.61)
6MWT	.239/0.174 (-0.21 - 0.68)	.211/0.231 (-0.23 - 0.62)	.460^**^/0.006 (0.15 - 0.79)	0.315/0.070 (-0.05 - 0.65)	.411^*^/0.016 (0.10 - 0.73)	.181/0.305 (-0.29 - 0.61)	1

* p < 0.05; ** p < 0.01 Pearson correlation test (Benjamini-Hochberg False Discovery Rate correction applied). The 95% confidence intervals (CI) represent the range within which the true correlation coefficient is likely to fall

SD: Standard Deviation; Pred.: Predicted; FVC: Forced Vital Capacity; FEV1: Forced Expiratory Volume in One Second; PEF: Peak Expiratory Flow; FEF25-75: Forced Expiratory Flow at 25-75% of Vital Capacity; MIP: Maximal Inspiratory Pressure; MEP: Maximal Expiratory Pressure; 6MWT: 6-Minute Walk Test

## Discussion

It is well established that respiratory functions are negatively impacted following OHS due to factors such as reduced lung compliance, atelectasis, sternotomy-related pain, and the potential for pneumonia [10, 11].

Previous studies consistently report significant decreases in spirometric values following open heart surgery. For instance, Urell C et al. [12] revealed substantial reductions in spirometric values two days post-operation compared to preoperative values. Similarly, Jasani N. et al. [13] reported significant postoperative declines in FEV_1_ and FVC five weeks post-surgery compared to their preoperative baselines. The most methodologically similar study to ours by Yuenyongchaiwat et al. [14] was a randomized controlled trial that compared the effects of a virtual reality (VR) exercise program versus conventional physical therapy during cardiac rehabilitation Phase I. This study also indicated significant postoperative declines in FVC and FEV_1_ at discharge compared to the preoperative period. Consistent with these findings, our study also observed significant reductions in FVC and 6MWT distance at discharge compared to preoperative values, as detailed in Table 3. However, FEV_1_ did not show a significant reduction.

This observed decline in pulmonary function can be attributed to several interacting mechanisms inherent to the post-cardiac surgery period ([10–11]). Post-sternotomy pain, for instance, severely restricts deep breathing and effective coughing, leading to shallow respiration (hypoventilation) and impaired clearance of airway secretions. This often precipitates atelectasis, where parts of the lung collapse due to insufficient air entry, further reducing lung volumes and compliance. Additionally, the use of sedatives and analgesics post-operatively can depress the respiratory drive, exacerbating hypoventilation and potentially contributing to lung injury. Furthermore, surgical procedures themselves and the effects of general anaesthesia can directly lead to pulmonary collapse and lung damage. Diaphragmatic dysfunction, which may occur due to phrenic nerve injury during surgery, can also contribute to this reduced inspiratory capacity [11]. Collectively, these acute post-surgical mechanisms significantly compromise gas exchange and overall pulmonary mechanics, providing a direct physiological explanation for the observed postoperative decrease in FVC (but not FEV_1_) in our patient cohort. Our discharge timing (9 ± 2 days) was consistent with that reported by Yuenyongchaiwat et al. [14]. Although our patients presented with lower preoperative pulmonary function values, their discharge values were comparatively better than those reported in some similar studies. This outcome was observed in patients who received a Phase I cardiac rehabilitation program as part of their routine postoperative care.

When reviewing the literature on the 6MWT used to assess functional capacity and exercise tolerance in patients following OHS, limited studies are available [15, 16]. Girgin et al. [15] investigated 6MWT distances in coronary artery bypass grafting (CABG) patients, reporting post-operative reductions in distance. Sheraz et al. [16] reported the minimum clinically important difference (MCID) for the 6MWT in CABG patients as 36 m. While they noted significant post-operative changes in distances at different time points, the difference between the third and seventh postoperative days, for example, was 28 m. This specific 28 m difference, being less than their reported MCID of 36 m, suggests it may not represent a meaningful clinical change despite any statistical significance. Chen et al. [17] similarly investigated 6MWT distances in OHS patients, reporting declines at discharge compared to preoperative values, followed by an improvement at three-month follow-up. Cordeiro et al. [18] also observed significant decreases in 6MWT distance at discharge in OHS patients. Consistent with these findings, our study also observed a significant decrease in preoperative 6MWT distance at discharge, as detailed in Table 3. This difference may be attributed to the early timing of the assessment at discharge and the varying severity of baseline characteristics.

Weak inspiratory muscle strength and functional capacity have been associated with adverse outcomes following OHS and are recognized as risk factors for postoperative morbidity and mortality [19]. For instance, Morsch et al. [20] reported a significant decline in both MIP and MEP by the sixth postoperative day in CABG patients. Yuenyongchaiwat et al. [14] similarly found significant declines in MIP and MEP at discharge. Conversely, another study found no significant changes in MIP and MEP at 8 weeks post-surgery compared to preoperative values [21]. In contrast to some of these studies, our findings indicate that there were no significant changes in mean MIP and MEP values between the preoperative period and discharge, as detailed in Table 3. It is important to note that the preoperative MIP values in our patient cohort were typically lower than those observed in healthy individuals, which is a common finding in patients undergoing open-heart surgery due to underlying cardiac and respiratory comorbidities [11]. Furthermore, our analysis revealed distinct patterns in inspiratory versus expiratory muscle function, with MEP values significantly higher than MIP values both preoperatively and at discharge (see Table 3 for details). Given the observed baseline differences, where MIP values were consistently lower than MEP values in our cohort, further research is warranted to investigate the specific role and benefits of targeted inspiratory muscle training within comprehensive cardiac rehabilitation programs following OHS.

The literature indicates that in healthy individuals, FEV_1_ is approximately 80% of FVC, a finding that is similarly observed in patients following OHS [22]. It has been reported that the relationship between PEF and FEV_1_ can vary, particularly depending on the severity of mild airway obstruction, suggesting a complex relationship between FVC, PEF, and FEV1 [23, 24]. In our study, the high correlation observed between PEF and FVC, and PEF and FEF25-75, provides valuable insights that may clarify the complex results reported in the literature. However, contrary to some literature, we found no significant correlation between FVC and FEV_1_, or between PEF and FEV_1_, in our cohort. Although not statistically significant (*p* = 0.058), we observed a slight increase in FEV_1_/FVC ratio from preoperative (77 ± 11%) to discharge (82 ± 18%) values (Table 3). This finding, while not indicative of airway obstruction resolution, is noteworthy given the overall pulmonary function decline and the smoking status of many of our patients.

The literature indicates a relationship between FVC and MIP in healthy individuals [25]. Similarly, Naseer et al. [21] reported a moderate correlation between MIP and forced expiratory volume in cardiac surgery patients during a two-month follow-up. While our study did not observe a statistically significant correlation between MIP and FVC, this finding suggests that in the early discharge phase post-OHS, the relationship between FVC and inspiratory muscle strength might be more complex or influenced by other factors such as pain, residual atelectasis, or differing recovery trajectories. The lack of statistical significance in our early discharge cohort, despite previous reports, highlights the importance of timing in assessing these relationships and may suggest that stronger correlations emerge later in the recovery process as acute post-surgical effects diminish.

Beyond isolated measures, our study significantly contributes to the literature by examining the interrelationships among inspiratory muscle strength, pulmonary function, and functional capacity outcomes in patients after OHS. Our analysis, which included Benjamini-Hochberg FDR correction, revealed significant positive correlations between MIP and 6MWT distance (*r* = 0.411, *p* = 0.016), as well as between FEV_1_ (% predicted) and 6MWT distance (*r* = 0.460, *p* = 0.006). These findings, detailed in Table 4, underscore a crucial interdependence between inspiratory muscle strength, specific aspects of respiratory function FEV_1_ (% predicted), and overall functional capacity following OHS. Therefore, in situations where direct assessment of functional capacity may be challenging in the early postoperative period, evaluating inspiratory muscle strength and pulmonary function could serve as valuable proxy measures, providing insights for rehabilitation and treatment planning.

### Strengths of the study

A significant strength of our study is the scarcity of research conducted on patients at the discharge phase following OHS, thus our results provide a valuable contribution to the existing literature.

### Limitations of the study

One limitation of our study is its single-center, retrospective design, which inherently limited the sample size to 34 patients, as it was dictated by the availability of complete patient records that met all specified inclusion criteria from the archives. Despite this relatively small number, the single-center approach allowed for consistency in the surgical procedures and rehabilitation applied. Another limitation is the diversity of surgical types included within the OHS cohort (e.g., CABG, valve surgery, ASD repair). This heterogeneity in surgical trauma, operative duration, and distinct recovery trajectories inherent to different procedures may confound the interpretation of overall respiratory and functional outcomes, as specific effects related to each surgical type could not be disaggregated or compared due to the sample size. Additionally, as a retrospective study, our dataset did not include systematically recorded specific post-operative respiratory complications, which limited our ability to perform predictive analyses such as sensitivity, specificity, and ROC curves to identify variables determining the risk for these complications.

This study, while providing valuable insights into early postoperative recovery, is not without limitations. A key limitation is its retrospective design, which restricted our ability to conduct long-term follow-up beyond the immediate discharge period. Given that the recovery of pulmonary function and functional capacity often evolves over several weeks and months post-surgery, the absence of longer-term data limits a comprehensive understanding of the full recovery trajectory. Future prospective studies with extended follow-up periods are essential to monitor these parameters over time and provide a more complete picture of recovery following open-heart surgery, potentially exploring the impact of continued outpatient rehabilitation.

## Conclusions

In patients undergoing OHS who received early physiotherapy, a significant reduction was observed in FVC and 6MWT distance at discharge compared to preoperative levels, while PEF, FEV1, FEF25-75, MIP, and MEP did not show significant changes. This study uniquely highlights the complex interrelationships among various physiological domains, demonstrating that decreased FVC and FEV_1_ at the discharge stage were specifically related to functional capacity. Therefore, routine assessment of respiratory muscle strength in OHS patients is essential, and these results, along with other respiratory function parameters, should be evaluated within homogeneous and specific surgical groups to guide effective rehabilitation and treatment planning.

## Data Availability

No datasets were generated or analysed during the current study.

## Abbreviations

6MWT: 6-Minute Walk Test
ATS: American Thoracic Society
ATS/ERS: American Thoracic Society/European Respiratory Society
CABG: Coronary Artery Bypass Graft
FDR: False Discovery Rate
FEF25-75: Forced Expiratory Flow at 25–75% of FVC
FEV_1_: Forced Expiratory Volume in One Second
FVC: Forced Vital Capacity
MCID: Minimum Clinically Important Difference
MEP: Maximal Expiratory Pressure
MIP: Maximal Inspiratory Pressure
OHS: Open Heart Surgery
PEF: Peak Expiratory Flow
VR: Virtual Reality
